# FOXD1 is a prognostic biomarker and correlated with macrophages infiltration in head and neck squamous cell carcinoma

**DOI:** 10.1042/BSR20202929

**Published:** 2021-07-02

**Authors:** Huazhen Liang, Chunning Zhang, Chaoming Li, Changguo Li, Yanli Wang, Huaming Lin

**Affiliations:** The First Tumor Department, Maoming People’s Hospital, Southern Medical University, Maoming, Guangdong 525000, China

**Keywords:** FOXD1, HNSC, immunosuppression status, Pan-cancer, TAM infiltration, TCGA

## Abstract

**Background:** Forkhead Box D1 (FOXD1) is differentially expressed in various tumors. However, its role and correlation with immune cell infiltration remains uncertain in head and neck squamous cell carcinoma (HNSC).

**Methods:** FOXD1 expression was analyzed in The Cancer Genome Atlas (TCGA) pan-cancer data. The clinical prognosis influence of FOXD1 was evaluated by clinical survival data of TCGA. Enrichment analysis of FOXD1 was performed using R packages ‘clusterProfiler’. We downloaded the immune cell infiltration score of TCGA samples from published articles, and analyzed the correlation between immune cell infiltration level and FOXD1 expression.

**Results:** FOXD1 was highly expressed and associated with poorer overall survival (OS, *P*<0.0001), disease-specific survival (DSS, *P*=0.00011), and progression-free interval (PFI, *P*<0.0001) in HNSC and some other tumors. In addition, FOXD1 expression was significantly correlated with infiltration of immune cells. Tumor-associated macrophages (TAMs) infiltration increased in tissues with high FOXD1 expression in HNSC. Immunosuppressive genes such as *PD-L1, IL-10, TGFB1*, and *TGFBR1* were significantly positively correlated with FOXD1.

**Conclusions:** Our study suggests FOXD1 to be an oncogene and act as an indicator of poor prognosis in HNSC. FOXD1 might contribute to the TAM infiltration in HNSC. High FOXD1 may be associated with tumor immunosuppression status.

## Introduction

Head and neck squamous cell carcinoma (HNSC) is one of the most common malignancies worldwide. It is prone to relapse and metastasis at the late stage, produced drug resistance and has a high mortality rate [1]. At present, there is still a lack of effective early tumor markers and diagnostic methods. With the rapid development of high-throughput sequencing technology and transcriptomics research, more and more key driver genes are discovered. However, there is still a clear need to identify key driver genes, especially genes that could affect the composition of the immune microenvironment in HNSC.

Forkhead Box D1 (FOXD1) plays an oncogene role in various tumor types. For example, FOXD1 is overexpressed in lung cancer and activate galectin-3/LGALS3 expression to promote lung cancer aggressiveness [2]. In addition, high FOXD1 expression in colorectal cancer is reported to predict poor survival of patients, FOXD1 promotes progression of colorectal cancer by activating ERK 1/2 pathway [3,4]. However, the role of FOXD1 in HNSC remains unknown.

In our study, we evaluated the expression of FOXD1 in various tumors in The Cancer Genome Atlas (TCGA) and its correlation with clinical features and prognosis of tumor patients. We found FOXD1 to be high-expressed in most tumors including HNSC. High FOXD1 expression was proven to associate with poorer overall survival (OS), disease-specific survival (DSS), and progression-free interval (PFI) of HNSC patients. FOXD1 was predicted to involve cell cycle-related pathways. As the infiltration of immune cells (especially macrophage) is important for the prognosis of HNSC patients [5,6], we examined the correlation between FOXD1 expression and immune cell infiltration score and found that tumor-associated macrophages (TAMs) infiltration significantly increased in tissues with high FOXD1 expression. Moreover, FOXD1 was positively correlated with immunosuppressive genes, such as *PD-L1, TGFB1*, and *TGFBR1*. Our results offer novel insights into the functional role of FOXD1 in HNSC, thereby highlighting a potential mechanistic basis whereby FOXD1 influences TAM infiltration and immunosuppressive gene expression in tumor microenvironment (TME).

## Materials and methods

### Human tissue samples

All experiments involving humans were in accordance with the principles of the Declaration of Helsinki, and approved by the Institutional Review Board of Maoming People’s Hospital of Guangdong, China. All subjects provided informed consent.

### Immunohistochemistry

Formalin-fixed paraffin-embedded tissues were cut into 4-μm sections. The sections were removed paraffin using xylene, and then immersed in the distilled water following routine methods. Antigen heat repair was performed using boiling 0.01 M citrate buffer. Afterwards, the sections were incubated with FOXD1/BF-2 antibody (ab129324, ABSci, China) for 12 h at 4°C. Then, these sections were rinsed in PBS three times and incubated with secondary antibody for 20 min at 37°C. Colouration with 3,3′-diaminobenzidine (DAB) was performed and followed by Hematoxylin staining. The sections were mounted with neutral gum and observed under microscope (Olympus).

### Data collection and analysis

FOXD1 expression profiles and clinical information of TCGA pan-cancer data were downloaded from UCSC Xena (https://xenabrowser.net/datapages/) database. TIMER 2.0 database (http://timer.cistrome.org/) were used to evaluate FOXD1 expression in TCGA pan-cancer. Full names of tumor abbreviation are placed in Supplementary Table S1.

### Correlation and enrichment analyses

The correlation analysis between FOXD1 and other mRNAs was performed in TCGA HNSC data, and Pearson correlation coefficient was calculated. The top 300 genes most positively associated with FOXD1 were selected into enrichment analysis to reflect the function of FOXD1. Gene Ontology (GO) and Kyoto Encyclopedia of Genes and Genomes (KEGG) analysis were performed using R package ‘clusterProfiler’, with the parameters: pvalue-Cutoff = 0.05, and qvalue-Cutoff = 0.05. Gene Set Enrichment Analysis (GSEA) was conducted using R package ‘clusterProfiler’, with the parameters: nPerm = 1000, minGSSize = 10, maxGSSize = 1000, and pvalue-Cutoff = 0.05.

### Immune cell infiltration

We downloaded the immune cell infiltration score of TCGA pan-cancer from the published study ‘The Immune Landscape of Cancer’ [7], in which immune cell infiltration score was estimated using CIBERSORT. Samples of TCGA HNSC were divided to two groups (including high-FOXD1 group and low-FOXD1 group) based on the median of FOXD1 expression to compare the level of immune cell infiltration.

### Tumor mutation burden calculation

TCGA somatic mutation was downloaded from UCSC XENA database. Tumor Mutation Burden (TMB) was calculated as the number of mutation base in per million bases based on somatic mutation data in each tumor from TCGA. The TMB results are shown in Supplementary Table S2.

## Results

### Pan-cancer FOXD1 expression analysis

**Figure 1:**
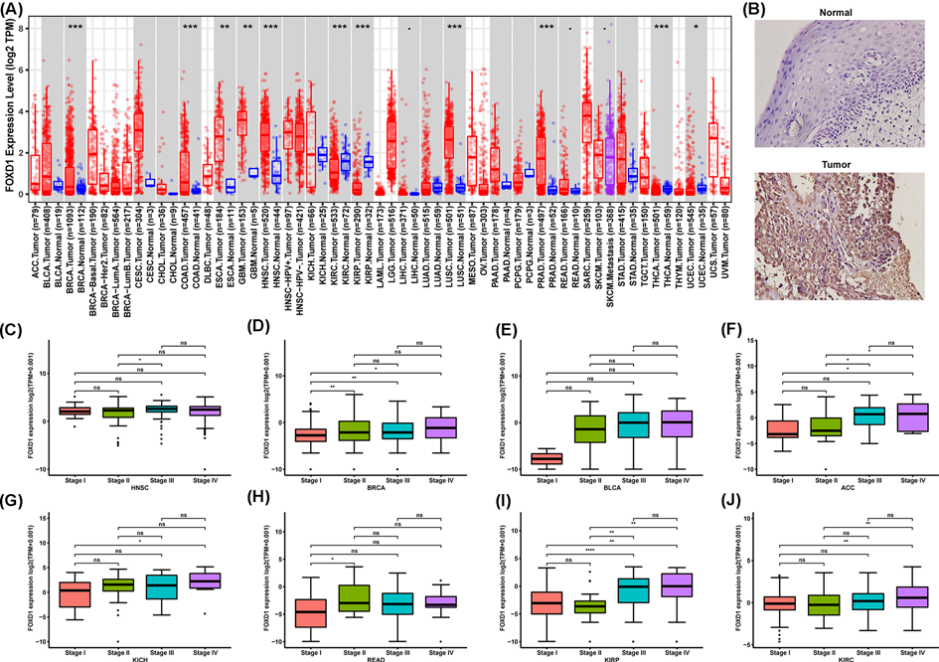
Pan-cancer FOXD1 expression analysis (**A**), FOXD1 expression in tumor and normal tissues in pan-cancer data of TCGA. (**B**) Representative immunohistochemical images of FOXD1 protein expression in tissues from oral squamous cell carcinoma and oral normal epithelium. (**C–J**), FOXD1 expression in different stages in indicated tumor type. Data were shown as mean ± SD. *P<0.05, **P<0.01, ***P<0.001, ****P<0.0001.

We first assessed the FOXD1 expression in pan-cancer data of TCGA. The analysis results revealed FOXD1 expression to be higher in seven tumors, including BRCA, COAD, ESCA, GBM, HNSC, LUSC and PRAD, while lower expression was observed in KIRC, KIRP, THCA and UCEC (Figure 1A). Immunohistochemistry revealed that FOXD1 protein level was higher in oral squamous cell carcinoma than that in normal oral epithelium (Figure 1B). In addition, the expression of FOXD1 was closely related to the clinical stage. FOXD1 expression was higher in patients with relatively high stages in several tumor types, including HNSC, BRCA, BLCA, ACC, KICH, READ, KIRP, and KIRC (Figure 1C–J). For paired tumors and normal tissues, FOXD1 was overexpressed in tumor tissues of HNSC, COAD, BRCA, CESC, STAD, BLCA, ESCA, CHOL, PRAD, LIHC, and LUSC (Supplementary Figure S1A–K), while low expressed in tumor tissues of KIRP, KIRC, and THCA (Supplementary Figure S1L–N).

### The association between FOXD1 expression and cancer patient prognosis

**Figure 2:**
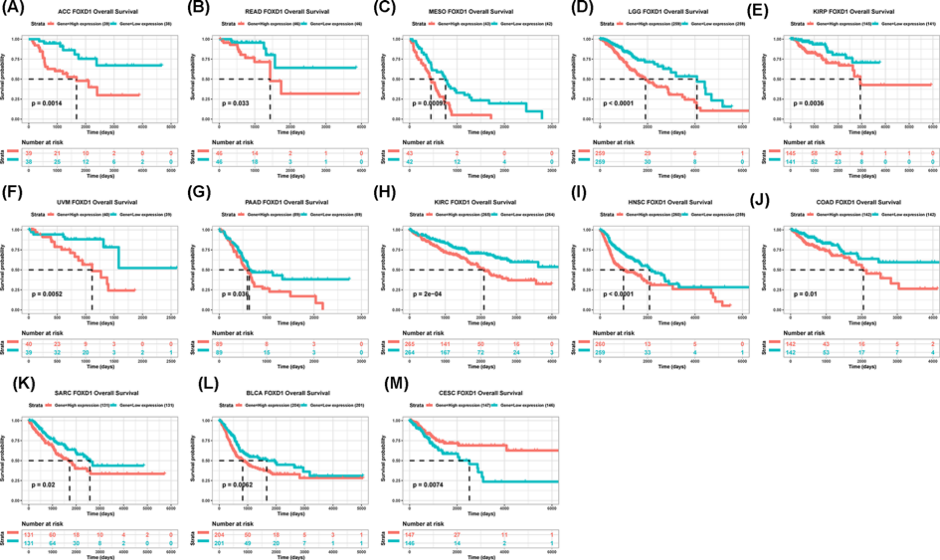
The association between FOXD1 expression and OS of cancer patients (**A–M**) Kaplan–Meier analysis of OS in 33 TCGA tumor types. Group division was based on the median of FOXD1 expression. Meaningless results were not shown.

**Figure 3:**
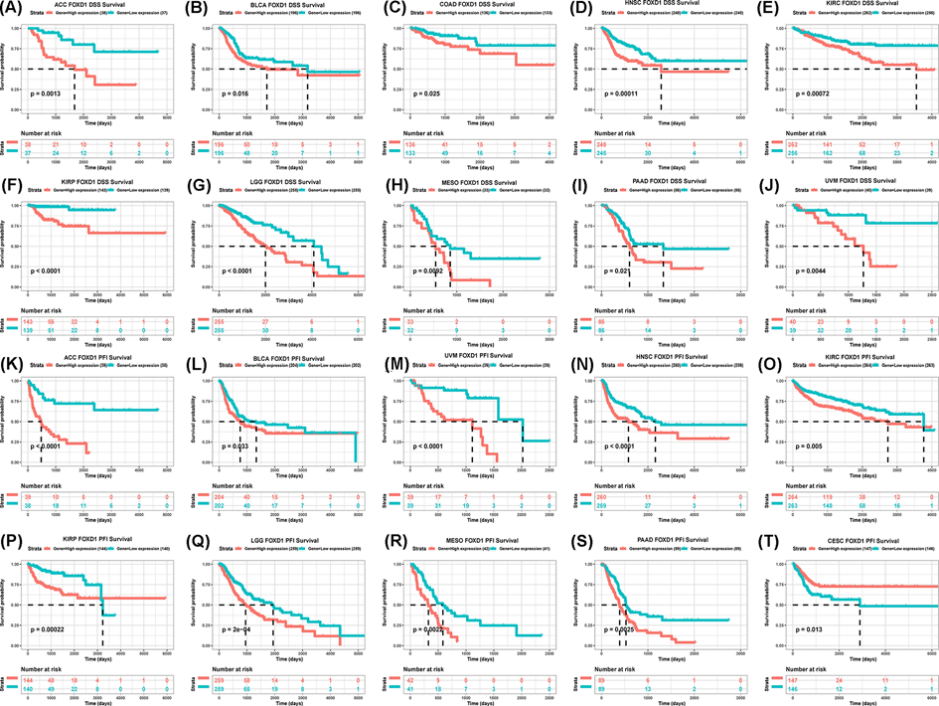
The association between FOXD1 expression and DSS and PFI of cancer patients (**A–J**) Kaplan–Meier analysis of DSS in 33 TCGA tumor types. group division was based on the median of FOXD1 expression. Meaningless results were not shown. (**K–T**) Kaplan–Meier analysis of PFI in 33 TCGA tumor types. Group division was based on the median of FOXD1 expression. Meaningless results were not shown.

To evaluate the value of FOXD1 in predicting the prognosis of tumor patients, the association between FOXD1 expression with OS, DSS, and PFI was analyzed in TCGA cohort. For OS, higher expression of FOXD1 was significantly associated with reduced OS time in the ACC (*P*=0.0014), READ (*P*=0.033), MESO (*P*=0.00097), LGG (*P*<0.0001), UVM (*P*=0.0052), PAAD (*P*=0.036), KIRC (*P*=0.0002), HNSC (*P*<0.0001), SARC (*P*=0.02), and BLCA (*P*=0.0062) (Figure 2A–L), while increasing OS time in the CESC (*P*=0.0074) (Figure 2M). For DSS, higher FOXD1 expression was significantly associated with reduced DSS in ACC (*P*=0.0013), BLCA (*P*=0.016), COAD (*P*=0.025), HNSC (*P*=0.00011), KIRC (*P*=0.00072), KIRP (*P*<0.0001), LGG (*P*<0.0001), MESO (*P*=0.00092), PAAD (*P*=0.021), and UVM (*P*=0.0044) (Figure 3A–J). In addition, the PFI reduced in high-FOXD1 expression group in ACC (*P*<0.0001), BLCA (*P*=0.033), HNSC (*P*<0.0001), KIRC (*P*=0.005), KIRP (*P*=0.00022), LGG (*P*=0.00002), MESO (*P*=0.0022), PAAD (*P*=0.0025), and UVM (*P*<0.0001) (Figure 3K–S), while increasing CESC (*P*=0.013) (Figure 3T). Since there were various tumor locations in HNSC, we further analyzed the OS in different tumor locations. The results revealed that high FOXD1 expression predicted worse OS especially in larynx tumor, hypopharynx tumor, and floor of mouth tumor (Supplementary Figure S2A–L).

### Correlation and enrichment analyses

**Figure 4:**
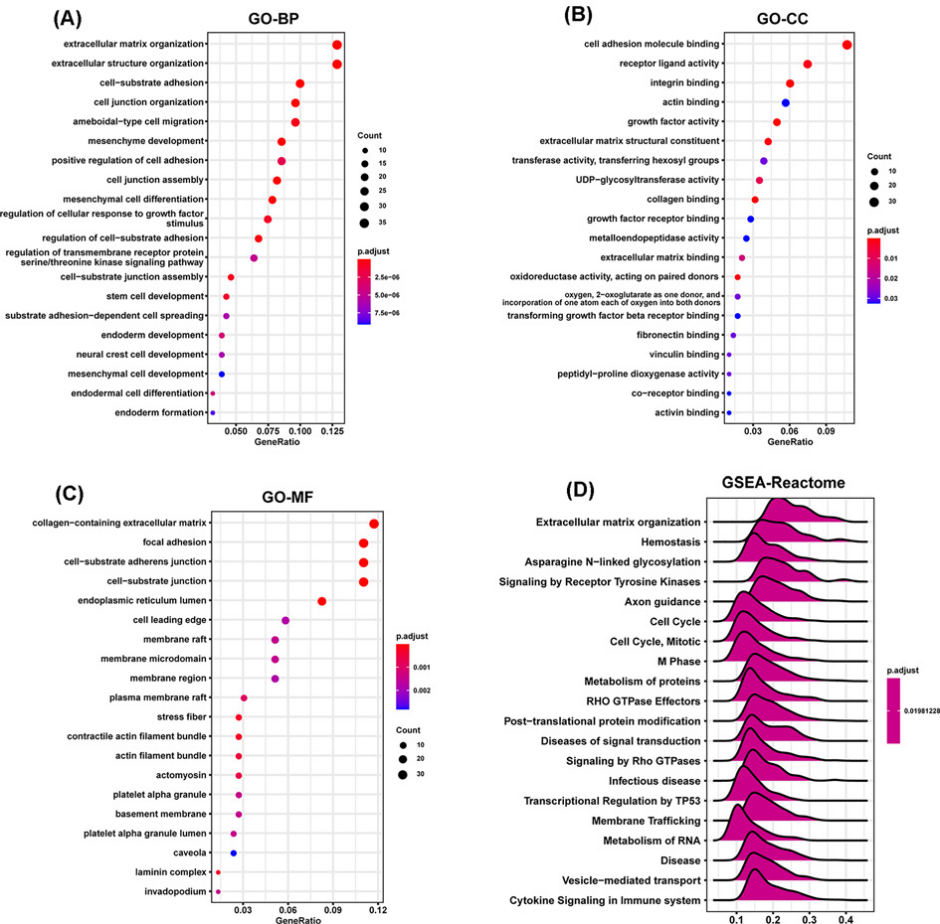
Function and pathway enrichment analysis of FOXD1 in HNSC (**A–C**) Significant GO terms of top 300 genes most positively associated with FOXD1, including BPs, MF, and CC in HNSC. (**D**) Significant GSEA results of FOXD1 in HNSC.

To predict the functions or pathways that FOXD1 may affect, we performed the correlation analysis between FOXD1 and other genes in HNSC using TCGA data (Supplementary Figure S3A,B). The top 300 genes most positively associated with FOXD1 were selected into enrichment analysis using R package ‘clusterProfiler’. The functional enrichment results revealed FOXD1 to be mainly associated with extracellular matrix organization in biological process (BP) category (Figure 4A), cell adhesion molecule binding in cellular component (CC) category (Figure 4B), and collagen-containing extracellular matrix in molecular function (MF) category (Figure 4C). GSEA was conducted to search Reactome Pathways FOXD1 may affect. The GSEA results showed that extracellular matrix organization and cell cycle pathways were significantly enriched (Figure 4D). These results suggested FOXD1 to be associated with many malignant pathways in HNSC, especially tumor invasion and proliferation-related pathways.

### The correlation analysis between immune cell infiltration and FOXD1

**Figure 5:**
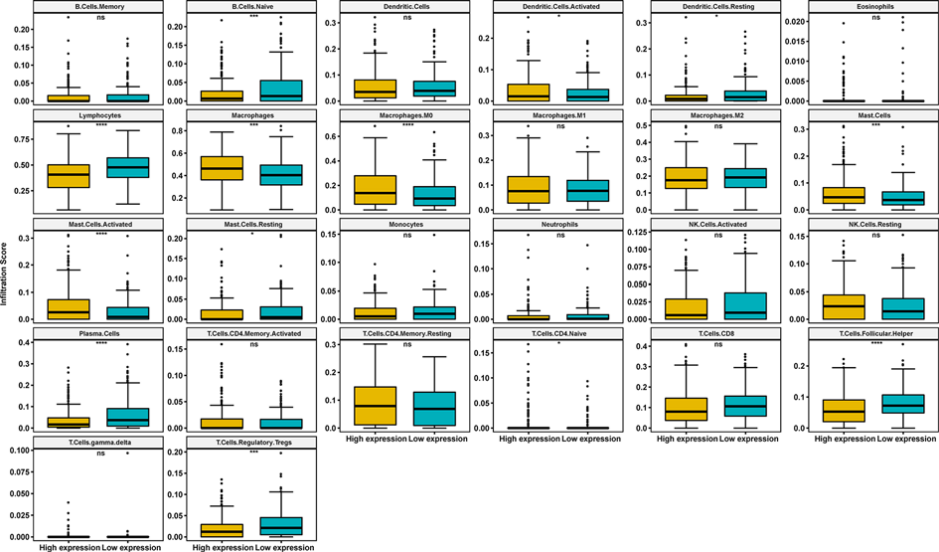
The difference analysis of immune cell infiltration in high- and low-FOXD1 expression groups The immune cell infiltration level in high-FOXD1 and low-FOXD1 expression group in HNSC of TCGA cohort. Data were shown as mean ± SD. *P<0.05, ***P<0.001, ****P<0.0001.

**Figure 6:**
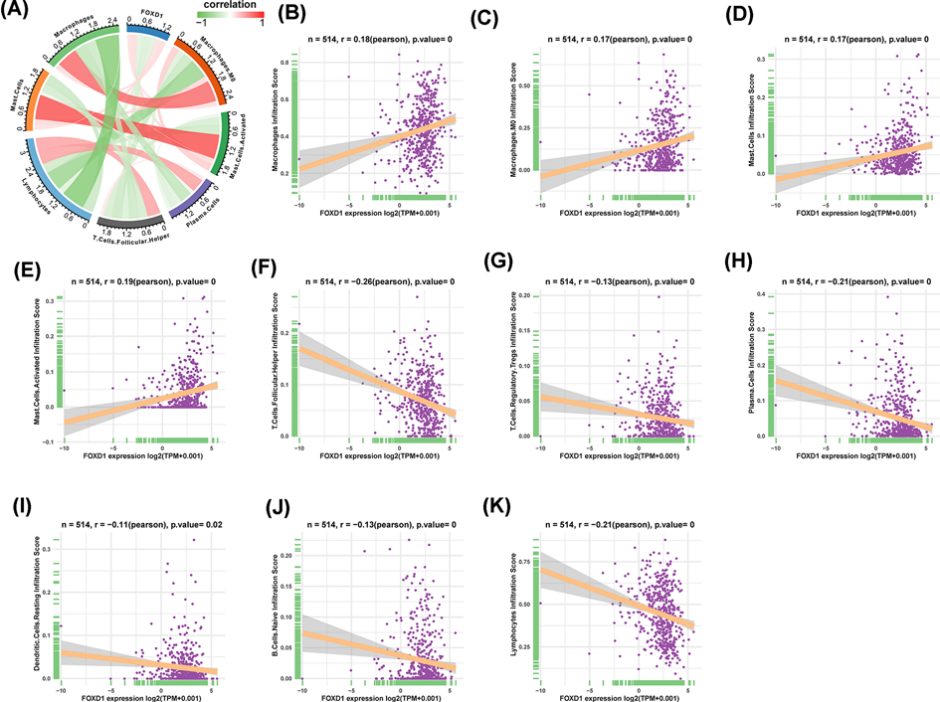
The correlation analysis between immune cell infiltration and FOXD1 in HNSC (**A**) The correlation between FOXD1 and relevant immune cells. Red lines represent positive correlation, green lines represent negative correlation; the deeper the color, the stronger the correlation. (**B–K**) Individual correlation analysis plots of FOXD1 and relevant immune cells.

**Figure 7:**
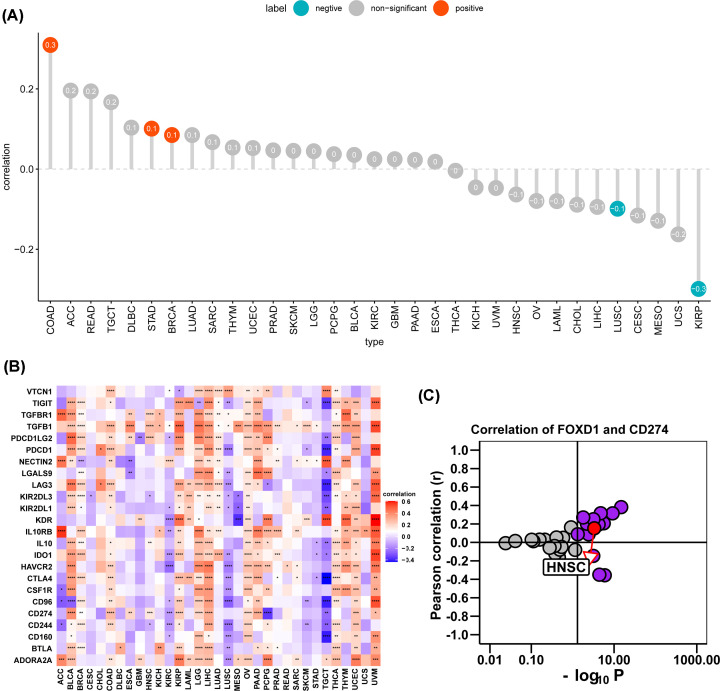
The effects of FOXD1 on immunosuppressive status in HNSC (**A**) The correlation between FOXD1 and TMB values in HNSC. Red circles represent positive correlation, cyan circles represent negative correlation, and gray circles mean no correlation. The number in the circle represents the correlation coefficient. (**B**) The correlation between FOXD1 and immunosuppressive genes was shown in heatmap. Red represents positive correlation, blue represents negative correlation; and the deeper the color, the stronger the correlation. Data were shown as mean ± SD. *P<0.05, **P<0.01, ***P<0.001, ****P<0.0001. (**C**) Correlation coefficient and −log10(*P*-value) of FOXD1 and CD274 are shown. Each circle represents a different tumor in TCGA. Red circle is marked for HNSC. Gray circles mean no correlation.

We further downloaded the immune cell infiltration score of TCGA HNSC. We noticed that macrophages and M0 macrophages infiltration level were higher in high-FOXD1 expression group (Figure 5). Correlation analysis revealed FOXD1 to be positively associated with infiltration level of macrophages, M0 macrophages, mast cells and activated mast cells, while negatively associated with infiltration level of follicular helper T cells, regulatory T cells tregs, plasma cells, resting dendritic cells, naive B cells, and lymphocytes (Figure 6A–K). TAM infiltration and TMB status are closely related to the immunosuppressive state of tumor [8]. We further analyzed the relationship of FOXD1 with TMB and immunosuppressive genes using TCGA pan-cancer data. As shown in Figure 7A, FOXD1 was positively correlated with TMB in COAD, STAD and BRCA, while negatively correlated with TMB in LUSC and KIRP. In addition, FOXD1 was positively associated with immunosuppressive genes, especially for PD-L1 (CD274), TGFB1 and TGFBR1, in most tumors, including HNSC (Figure 7B,C). These results suggest that the high expression of FOXD1 is closely related to the immunosuppressive status of HNSC.

## Discussion

FOXD1 is involved in the progression of many diseases, including several tumor types [9–11]. Previous studies have shown the oncogenic role of FOXD1 in glioma [12], non-small cell lung cancer [13], and breast cancer [14] etc. FOXD1 has not been extensively studied, especially in HNSC. Lin et al. reported that knockdown of FOXD1 could potentiate radiation effectiveness by down-regulating G3BP2 expression and promoting the activation of TXNIP-related pathways in oral cancer, indicating that targeting FOXD1 might be a good strategy to enhance the radiosensitivity of oral cancer [15]. This study provides us the evidence that FOXD1 promotes oral cancer progression. However, more evidence should be explored. Therefore, it is urgent to clarify the role of FOXD1 in HNSC progress and treatment.

In our study, we examined the FOXD1 expression levels and prognostic function in pan-cancer using TCGA data from UCSC Xena. Based on our results, we found FOXD1, compared with normal tissues, to be overexpressed in BRCA, COAD, ESCA, GBM, HNSC, LUSC, and PRAD, while lower expression was observed in KIRC, KIRP, THCA, and UCEC. The difference of FOXD1 expression levels in different tumors types may reflect the distinct underlying functions and mechanisms. We further found that overexpression of FOXD1 generally predicts poor prognosis in tumors with high FOXD1 expression, such as ACC, READ, MESO, LGG, UVM, PAAD, KIRC, HNSC, SARC, and BLCA. In contrast, low FOXD1 expression indicates poor prognosis in CESC. These results indicate that FOXD1 could be a prognostic biomarker to predict the prognosis of tumor patients.

TME cells, especially immune microenvironment, constitute a vital element of tumor tissue. Increasing evidence has revealed their clinicopathological significance in predicting outcomes and therapeutic efficacy [16,17]. The infiltration of TAM facilitates the progression of epithelial HNSC [18,19]. Our results proved that FOXD1 to have close relationship with TAM infiltration. TAM infiltration level was significantly higher in high FOXD1 expression group. Moreover, the positive correlation between FOXD1 expression and immunosuppressive genes, such as *PD-L1, IL-10, TGFB1* and *TGFBR1*, indicate the key role of FOXD1 in regulating tumor immunology. The high expression of FOXD1 indicates the immunosuppression of HNSC, providing a potential target and clue for immunotherapy of HNSC.

In summary, FOXD1 may play an important role in immunosuppressive state of HNSC and act as a valuable prognostic biomarker in HNSC.

## Supplementary Material

Supplementary Figures S1-S3

Supplementary Tables S1-S2

## Data Availability

All the data will be provided on reasonable request from the corresponding author.

## Abbreviations

DSS: disease-specific survival
FOXD1: Forkhead Box D1
GSEA: Gene Set Enrichment Analysis
G3BP2: GTPase activating protein (SH3 domain) binding protein 2
HNSC: head and neck squamous cell carcinoma
IL: Interleukin
OS: overall survival
PFI: progression-free interval
TAM: tumor-associated macrophage
TCGA: The Cancer Genome Atlas
TMB: tumor mutation burden
TME: tumor microenvironment
TXNIP: Thioredoxin interacting protein
