# Supplementary material for: FOXD1 is a prognostic biomarker and correlated with macrophages infiltration in head and neck squamous cell carcinoma
Source: Biosci Rep. 2021 Jul 2;41(7):BSR20202929. doi: 10.1042/BSR20202929 (PMC8255535; doi:10.1042/BSR20202929)
Supplement: Supplementary Figures S1-S3 [file BSR-2020-2929_supp.pdf]

Supplementary figure legends

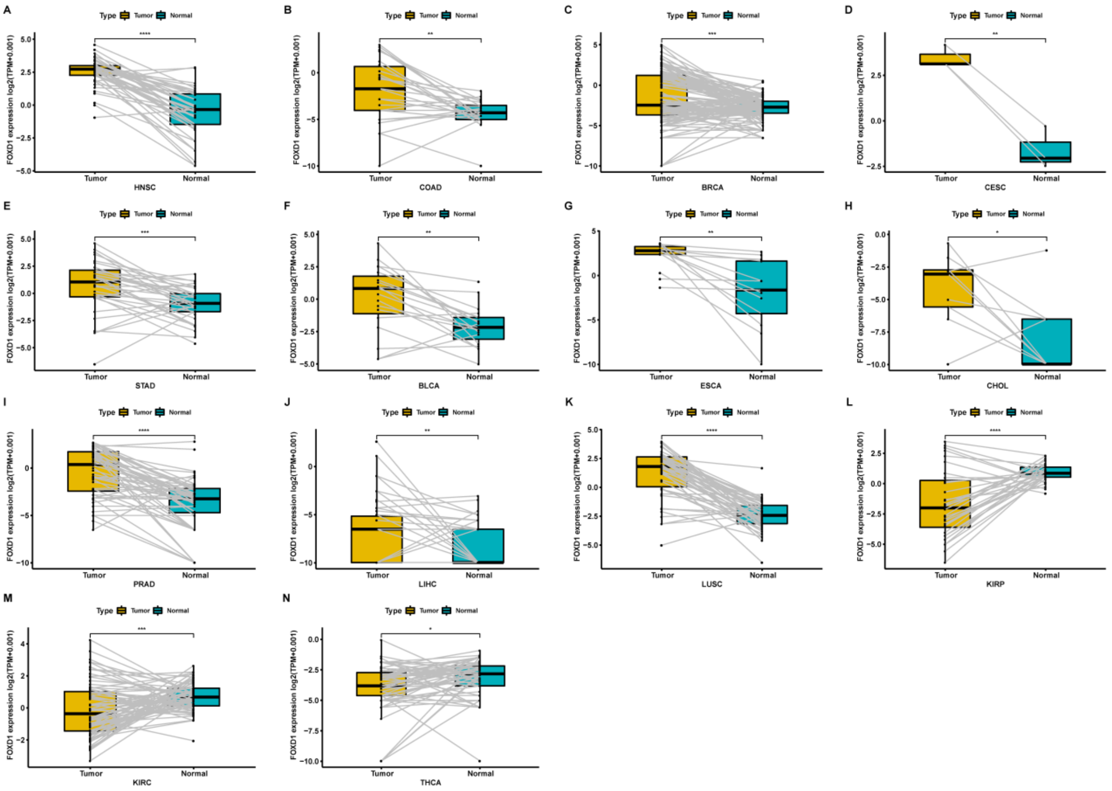

Sup-Figure 1: Pan-cancer FOXD1 expression analysis in paired tumor and normal tissues in TCGA. (**A-N**), FOXD1 expression in indicated paired tumor and normal tissues in pan-cancer data of TCGA. Gray lines connect paired tissues. Data were shown as mean ± SD. *$p < 0.05$, **$p < 0.01$, ***$p < 0.001$, ****$p < 0.0001$.

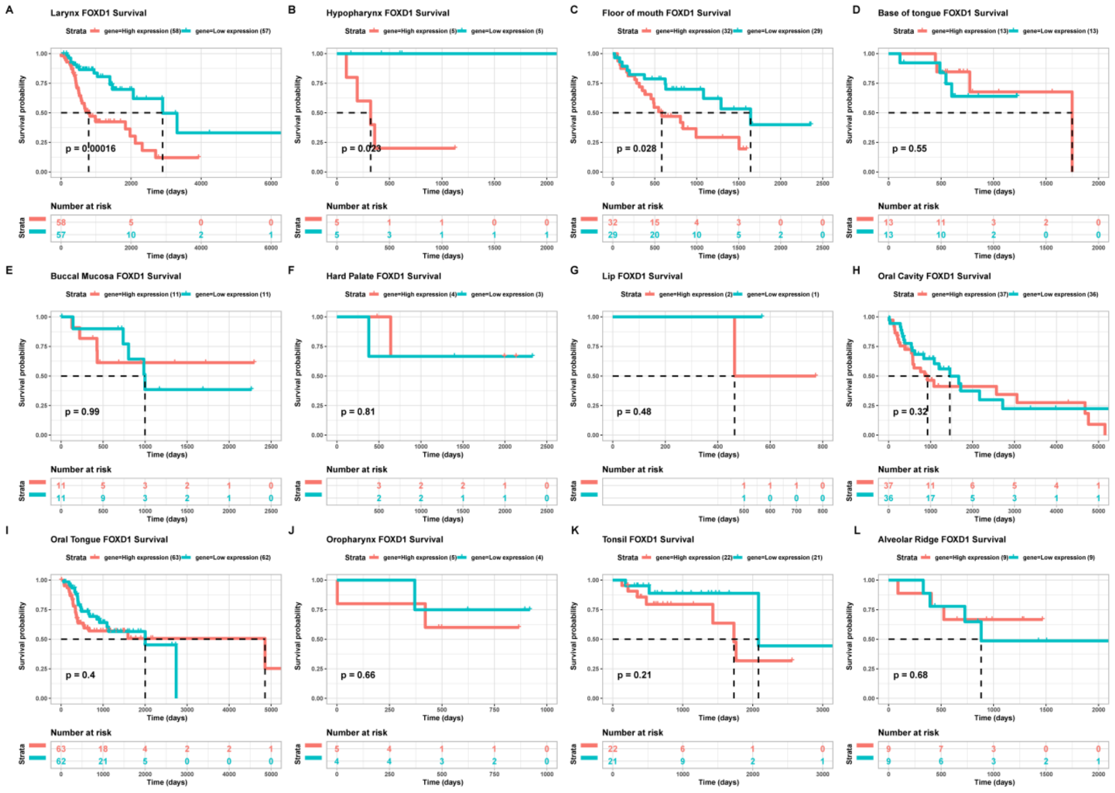

Sup-Figure 2: Correlation analysis of FOXD1 in HNSC.

(**A-L**) Kaplan–Meier analysis of OS in 16 various tumor locations of HNSC, group division was based on the median of FOXD1 expression.

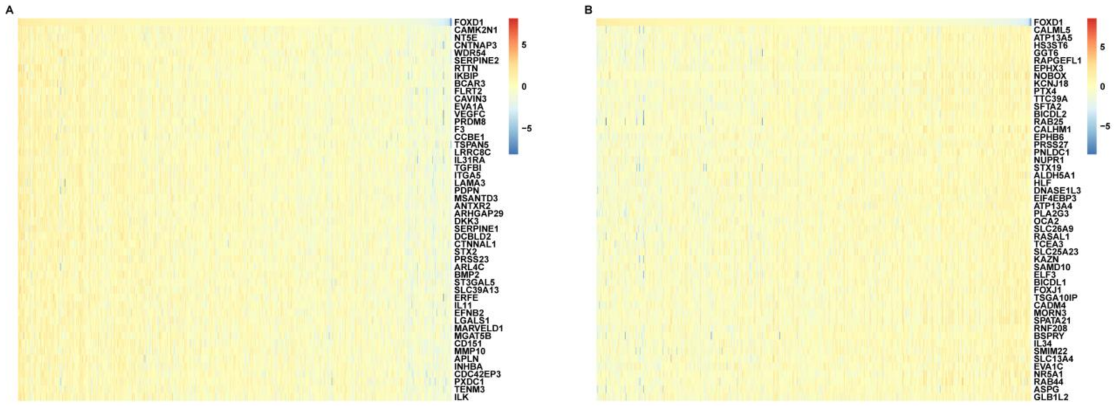

Sup-Figure 3: Correlation analysis of FOXD1 in HNSC.

(**A**), Top 50 genes most positively associated with FOXD1 were shown in heatmap.

(**B**), Top 50 genes most negatively associated with FOXD1 were shown in heatmap.
